# Evaluating the value of day 0 of an ICSI cycle on indicating laboratory outcome

**DOI:** 10.1038/s41598-020-75164-9

**Published:** 2020-11-09

**Authors:** E. Maziotis, K. Sfakianoudis, P. Giannelou, S. Grigoriadis, A. Rapani, P. Tsioulou, K. Nikolettos, A. Pantou, A. Tiptiri-Kourpeti, M. Koutsilieris, B. Asimakopoulos, N. Nikolettos, K. Pantos, Mara Simopoulou

**Affiliations:** 1 Department of Physiology, Medical School, National and Kapodistrian University of Athens, Athens, Greece; 2 Department of Physiology, Medical School, Democritus University of Thrace, Alexandroupoli, Greece; 3 Centre for Human Reproduction, Genesis Athens Clinic, Athens, Greece; 4 Assisted Reproduction Unit of Thrace “Embryokosmogenesis”, Alexandroupoli, Greece; 5 Assisted Reproduction Unit, 2nd Department of Obstetrics and Gynaecology, Aretaieion Hospital, National and Kapodistrian University of Athens, 76, Leof. Vasilissis Sofias, 11526 Athens, Greece

**Keywords:** Embryogenesis, Embryology

## Abstract

A number of oocyte characteristics have been associated with fertilization, implantation and live-birth rates, albeit without reaching a consensus. This study aims to delineate possible associations between oocyte characteristics, oocyte behavior during intracytoplasmic sperm injection (ICSI), fertilization potential, and laboratory outcomes. Four-hundred and seventy-seven patients, yielding 3452 oocytes, were enrolled in this prospective observational study from 2015 to 2018. Οoplasm granularity was associated with poor embryo quality and higher probabilities of post-ICSI oocytes and embryos discarded in any developmental stage and never selected for embryo transfer or cryopreservation (*p* < 0.001). Both sudden or difficult ooplasm aspiration, and high or lack of resistance during ICSI were associated with either a poor Zygote-Score or fertilization failure (*p* < 0.001). Sudden or difficult ooplasm aspiration and high resistance during ICSI penetration were positively associated with resulting to a post-ICSI oocyte or embryo that would be selected for discard. Evaluation of oocyte characteristics and oocyte behavior during ICSI may provide early information regarding laboratory and cycle outcomes. Particularly, ooplasm granularity, and fragmentation of polar body, along with sudden or difficult ooplasm aspiration and high or lack of resistance during ICSI penetration may hinder the outcome of an ICSI cycle. The associations presented herein may contribute towards development of a grading system or a prediction model. Taking into account information on oocytes and ICSI behavior may effectively assist in enhancing IVF outcome rates.

## Introduction

In vitro fertilization (IVF), entering the fifth decade of clinical practice from 1978 to the era of precision medicine, is a field of dynamic research focusing on continuous improvement. Numerous studies from embryo morphology to novel biomarkers have been introduced as the next holy grail in efficient embryo selection. The majority of the studies focus principally on the events that follow successful fertilization of the oocyte both in terms of morphology and morphokinetics^1,2^. Morphological evaluation of zygotes, cleavage stage embryos and blastocysts has been thoroughly investigated and a consensus was achieved for each of these stages in 2010 according to SART and 2011 according to ESHRE^3,4^. Still, to date there is not a universal way of classifying embryos. Since then, prediction models have been implemented^5^ and the introduction of Time-Lapse Microscopy (TLM) has contributed towards the development of further advanced grading systems relying on morphokinetics^6,7^. Interestingly, a consensus encompassing recent advancements has yet to be reached.

Gamete evaluation follows a considerably different trail. In most embryological data studies, evaluation and description is based commonly on reporting on oocyte yield and maturity status. Certain attempts have been made to suggest an oocyte grading system^8^. Nonetheless, in absence of a widely accepted grading system^9^, reporting on specific morphological parameters regarding the oocyte in relation to embryo data, may not be the standard in publications. Certain oocyte features have been successfully associated with fertilization outcome, zygote formation, embryonic development and implantation potential^10^. Documented studied oocyte parameters include the cumulus oocyte complex (COC)^11^, the polar body^12^, the zona pellucida (ZP)^13^, the perivitelline space (PVS)^14^ and the ooplasm^15^. In most cases the oocyte features were regarded as a dichotomous variable^15^ and not a multifactorial or continuous as they present in reality. Hitherto, only a single systematic review has been performed on the predictive value of the oocyte characteristics reaching the conclusion that data were contradicting and further research was required^9^.

Intracytoplasmic Sperm Injection (ICSI) is described as an invasive insemination method, that changed the reality and potential in ART by enabling azoospermic men to father children^16^. From employing ICSI on the grounds of male factor infertility associated with a poor semen analysis sample, to opting for ICSI solely on the grounds that it provides higher fertilization rates^17^, ICSI certainly remains a topic of extensive research. As a result, different schools of thought have been developed regarding ICSI practice and outcome. ICSI has been thoroughly studied investigating the possibility of additional risks in the prevalence of chromosomal^18^ and congenital malformations^19^ to obstetric, perinatal and neonatal complications. A recent metanalysis reported poorer metabolic and reproductive outcome of the offspring^20^. Breaking down ICSI practice, the oocyte’s behavior has been documented. As anticipated, oocyte behavior during ICSI has been observed to result in different outcomes regarding the developmental and the implantation potential of the embryo^21^. Besides morphology, behavior of the oocytes during ICSI is a topic that bibliographically has not yet claimed the attention it deserves, even though there are data that highlight its importance. The oolemma breakage^22,23^ is referred to employing various descriptions and evaluations^22,23^ The level of resistance met during penetration by the ICSI needle^24^ corresponding to the respective oolemma response, along with the ooplasm’s response during aspiration^24,25^, both serve as parameters defining oolemma breakage type and affecting subsequent development^24^. Interestingly, very limited data is available on either aspect of the oocyte’s behavior during ICSI, rendering the present study timely and essential.

The formation and morphological characteristics of the resulting zygote appear to be of importance regarding the developmental potential of the preimplantation embryo. Interestingly, recent studies revisiting the zygote score (Z-Score) scenario encompassed in TLM practice have indicated the importance of zygotes’ scoring, both in terms of the time of pronuclei (PN) formation^26^, PN disappearance^27,28^ as well as the juxtaposition area of the PNs^2^. A good quality zygote seems to be unanimously acknowledged to result to an optimal IVF outcome^3^.

It appears that in the maze of data aligned towards establishing a model to predict the embryo of the highest implantation potential, leading to the birth of a healthy child coupled by a reassuring pediatric follow up, perhaps certain aspects have remained understudied or underrepresented in literature. In light of that, it may be time for studies to focus on connecting the dots between the oocyte, its behavior during ICSI, embryo quality and live-birth potential. These early cycle data are not yet encompassed in any of the recent advancements in the field of IVF. It is this rational that fueled design of the present study. Hereby the authors proceed with providing data aiming to associate these levels namely the quality of oocyte retrieved, the subsequent ICSI application, the respective embryo quality and the cycle outcome. These different studied levels correspond to different timepoints in clinical practice. Overall, the present study aims to investigate possible associations between oocyte morphology and behavior during ICSI, with the fertilization outcome, the resulting zygote and embryo quality, and the implantation and live birth rates. Delineating on the effects of different features regarding oocyte morphology and relating them to the oocyte’s behavior during ICSI while completing this course including data on the resulting zygote, in light of the IVF cycle outcome, may subsequently assist the practitioners on understanding factors entailed in securing creation of a potent zygote and embryo.

## Materials and methods

A total of 477 couples who underwent 477 ICSI cycles, yielding a total of 3452 oocytes, participated in this prospective observational study from 2015 to 2018. This study was approved by the Clinic’s Ethics Review Board and is in accordance to the declaration of Helsinki. Couples evaluated as eligible for participation were provided adequate time to read and understand the informed consent form and were encouraged to ask the treating physician any emerging questions. It was thoroughly explained that the present study was of observational nature and participation would not require any alteration to the clinical standard operating procedure. The informed consent form specified that their anonymity would be ensured throughout the conduct of the study, as well as in the case of dissemination and publication of respective data. All participants were provided with two copies of the informed consent form and returned a signed copy. ICSI was performed on the grounds of male infertility. All couples included presented solely with male factor infertility, serving as the indication for performing ICSI. Couples with azoospermia or teratozoospermia were excluded as this was identified in potentially serving as a confounder regarding fertilization success^29^. Exclusion criteria for this study include diagnosis of endometriosis, sexually transmitted diseases (STDs), current or previous cancer diagnosis, genetic or endocrinologic disorders, as well as female factor infertility, described as abnormal parameters indicated and diagnosed during the basic infertility investigation. All couples underwent basic infertility investigation. Inclusion criteria for the present study were female age between 30 and 39 years of age, and male factor infertility attributed to oligozoospermia or asthenozoospermia or combination of them.

### Basic infertility investigation

Basic infertility investigation was performed as previously described^30^. Briefly, it included semen analysis, hysterosalpingography in order to evaluate patency of the fallopian tubes, and assessment of ovulatory function via evaluating FSH levels, LH levels, estradiol (E2) levels, and progesterone’s levels during the menstrual cycle combined with ultrasound screening. All study participants were ovulating normally and reported regular length of menstrual cycles ranging from 24 to 35 days. The exclusion criteria were abnormal karyotypes, FSH > 12 mIU/mL, LH > 12 mIU/mL, AMH < 1.1 ng/mL, age > 40 years old. Any abnormality observed in the anatomy of uterine cavity or functionality of the fallopian tubes as assessed by hysterosalpingography, lead to exclusion from the present study. Body Mass Index (BMI) above 30 or less than 18.5, Polycystic Ovarian Syndrome (PCOS), Premature Ovarian Insufficiency (POI) and poor ovarian response were further considered as exclusion factors.

### Controlled ovarian stimulation

Controlled ovarian stimulation was achieved employing the same protocol for all recruited patients. The Controlled Ovarian Stimulation protocol was the standard Gonadotropin-Releasing Hormone (GnRH) long agonist protocol, as previously described^31^. Briefly, the initiation of the protocol included administration of 0.1 mg GnRH agonist on day 21 of the previous cycle. Administration of gonadotropin at 300 international units (IU) was employed in a daily dose. The adjustment of gonadotropin dose was based on ultrasonographical assessment of follicular development. When one or more follicles reached a diameter ≥ 17–18 mm ovulation was triggered employing human chorionic gonadotrophin (hCG) administration. Serial determinations of serum estrogen (E2) and progesterone levels were performed during the treatment as well as ultrasound observation of the follicles. Transvaginal oocyte aspiration was performed 36 h post hCG administration.

### Single oocyte and embryo culture tracking system

The individualized oocyte and embryo culture tracking system employed for this study allowed the observer to track confidently each oocyte’s progress. The 60 mm petri dishes employed included 8 pre-aliquoted peripherally placed droplets of Universal IVF Medium (Origio), and a centrally placed wash-droplet overlaid with oil for tissue culture (Cooper Surgical). The dishes that were accommodating COCs, denuded oocytes, and inseminated oocytes following ICSI that were employed in the IVF laboratory on the day of oocyte retrieval (D0) were all prenumbered. Naturally, at each progressing stage from COCs, to MIIs subjected to ICSI, the number of occupied droplets per respective dish could be reduced. Nonetheless, as prenumbered dishes were employed during D0 to ascertain consistency, the unoccupied droplets’ numbering was “crossed-out” at the transfer time to avoid confusion. The dishes accommodating normally fertilized oocytes on Day 1 and embryos on Day 2 onwards were pre-aliquoted but not prenumbered. Following fertilization assessment only the identify numbers of the oocytes that were normally fertilized were represented and marked the respective droplets of the dish at the transfer time.

### Oocyte morphological evaluation, ICSI behavior assessment, Z-Score and embryo grading

The data presented herein document the studied parameters at several time-points as follows: COC morphological evaluation following retrieval at 36 h post hCG administration, oocyte morphological evaluation following denudation at 38 h, description of the oocytes’ behavior during ICSI, regarding prenetration difficulty and the subsequent ooplasm aspiration difficulty at 40 h post HCG injection, Z-score analysis of the zygotes, as developed by Scott et al.^32^ at 16–18 h post insemination, as well as embryo quality on day 3 according to Veeck et al.^33^. The oocytes were evaluated reporting on the COC, their size, shape, ooplasm characteristics, PVS, PB and ZP. The COC was evaluated as normal, abnormal or extensively abnormal. Evaluation of the COCs was performed following oocyte retrieval at the dissecting microscope. The classification employed describing the COC as normal, abnormal or extensively abnormal, referred and accounted on the expansion of the cumulus complex documented according to number of layers^34^, the architectural arrangement of cells describing tight or dense complexes^34^ corresponding to darker areas, whereas the presence of blood clots indicated another abnormal characteristic of the complexes as per Ebner et al.^11^. The remaining oocyte characteristics were evaluated following oocyte denudation at an inverted microscope. Oocytes were categorized according to size as small, normal or large. The oocytes of a diameter < 120 μm were classified as small, the oocytes of a diameter between 160 and 180 μm were classified as large. The diameter sizes employed for this classification are previously described by Lazzaroni-Tealdi and colleagues^8^. Diploid giant oocytes generally known to be referred to as twice the volume of a normal oocyte and of a diameter > 200 μm^35^ were in fact not encountered at all during the course of this study. The shape of the oocyte was evaluated as normal, ovoid, uneven or distorted. Elongated oocytes or other shape abnormalities were regarded as distorted. The ooplasm was evaluated as dark, granular or vacuolated. The ooplasm was noted as granular if granules were present at the center of the ooplasm, as described by Serhal^36^. Translucency of the ooplasm was also evaluated. The PVS was classified as normal, granular, large or absent. The ZP was assessed regarding both its size, as thin, normal or thick and its texture, as uneven, dark, granular or normal. Finally, the PB was categorized as normal, small, large, flat or fragmented.

ICSI behavior documentation pertained to assessing penetration difficulty classified as “no resistance”, “normal”, or “high resistance” as experienced by the practiotiner. Subsequent ooplasm aspiration categorization was described as “normal”, “sudden” or “difficult aspiration” as experienced by the practitioner. Regarding penetration resistance documentation, “no resistance penetration” describes the injection pipette entering the oocyte at 3 o’clock, puncturing the oocyte and starting to indent the membrane, but then instead of causing the membrane to form a funnel shape all the way until almost the middle of the oocyte’s diameter before the oolemma breaks-being the case for “normal resistance” as described by Palermo et al.^23^,-the injection needle enters the oolema suddenly, causing its breakage abruptly without any resistance met by the practitioner. “High resistance penetration” is documented when the penetrating injection pipette causes a funnel shape of the ooplasm all the way until almost the other side of the puncture site at 9 o’clock before oolemma breakage is achieved. “High resistance penetration” translates to the oocyte being subjected to prolonged pressure by the injection needle starting at the puncture site, and it corresponds to the practitioner experiencing this resistance met by the ooplasm, while the level of rigidity and elasticity of the membrane play a major role in this. Oolemma breakage types have been reported to be determined by taking into account both penetration type, along with suction/aspiration mode of the ooplasm during ICSI as described by Nagy and colleagues reporting on five different oolema breakage types^22^. Ooplasm aspiration into the injection pipette has been shown to ensure oolemma breakage according to Vanderzwalmen et al., 1996 and enhance oocyte activation^37^, while Rosen et al. support that free-flow of the ooplasm into the injection pipette confirms the former^38,39^. In this study ooplasm aspiration was categorized as “sudden”, “normal” and “difficult”. Following penetration of the injection pipette into the ooplasm, “difficult ooplasm aspiration” refers to the difficulty showcased by the ooplasm to be released in the injection pipette. This corresponds to a difficulty experienced by the practitioner while aspirating the ooplasm. Difficult ooplasm aspiration translates to the oocyte being subjected to prolonged suction until the ooplasm is finally released in the pipette. Sudden ooplasm aspiration is usually accompanied by an abrupt, easy aspiration and quick-flow of the ooplasm in the injection pipette without the resistance that is normally anticipated.

To ensure standardization in assessment of ICSI behaviour-being an admittedly subjective matter-ICSI for this study was performed by the same experienced HFEA licensed, ESHRE certified senior clinical embryologist. The same type of injection pipettes were employed, and oocyte penetration was performed in the same manner, hence it was the particular membrane’s and the ooplasms’s structure that defined the respective penetration and subsequent ooplasm aspiration type. Evaluations at all stages prior to and following insemination-namely oocyte grading, fertilization assessment, zygote and embryo scoring- were similarly performed by the same practitioner. The appointed practitioner was assisted during all stages by reporting to another clinical embryologist involved in simultaneously recording data.

Sixteen to 18 h post ICSI fertilization evaluation along with zygote scoring was performed. The zygote scoring was performed according to Scott^32^ (Supplementary Table 1). The authors classified the oocytes as unfertilized when featuring 0PN. 2PN zygotes that failed to cleave were categorized as uncleaved zygotes. Arrested embryos. Arrested embryos are described in this study as day 2 cleavage stage embryos that failed to progress any further with blastomeres’ not subsequent dividing as evident on day 3.

All women underwent double embryo transfer on Day 3 following single embryo culture. The two best Day 3 graded embryos were transferred. Day 3 embryo grading was performed according to Veeck’s grading system^33^. According to Veeck as grade 1 are categorized the embryos presenting with blastomeres of equal size and no fragmentation. Grade 2 embryos feature equal size blastomeres and up to 10% fragmentation. Grade 3 embryos present with unequal size blastomeres. Grade 4 embryos feature equal or unequal sized blastomeres and fragmentation between 10 and 50%, while grade 5 embryos present with more than 50% fragmentation.

### Statistical analysis

The statistical analysis was performed employing IBM SPSS Statistics (v.25) and R programming language. To evaluate possible associations between oocyte and patient characteristics Kendall’s tau was employed. In order for this association to be independent of the absolute number of oocyte yield, the association was performed between patient characteristics -namely age, AMH, FSH, E2, LH, and progesterone levels- with the observation frequency for each type of oocyte characteristic. To study a possible negative effect of oocyte characteristics and their behavior during ICSI on the cycle outcome, an analysis was performed regarding the aforementioned parameters and the post-ICSI oocytes and embryos that were discarded and not selected for embryo transfer or cryopreservation. This category of post-ICSI oocytes and embryos selected to be discarded includes unfertilized or abnormally fertilized oocytes, uncleaved zygotes, arrested day 2 embryos, and embryos that were categorized as grade 5 on day 3. To assess possible associations between oocyte morphology, behavior during ICSI and Z-Score the multinomial logistic regression was employed. Multinomial logistic regression was also employed regarding the associations between oocyte morphology and the respective ICSI behavior, with embryo grading on day 3, as well as with post-ICSI oocytes and embryos selected to be discarded. The “normal” feature of each parameter was employed as the reference point for the regression analysis. This enabled analysis encompassing the numerous associations studied herein. Regarding the Z-Score the Z1 was employed as the reference point. Regarding day 3 embryo quality grade 1 was regarded as the reference point. The initial significance level was set at *p* = 0.05. To eliminate possible multiple comparison bias regarding oocyte characteristics, a sensitivity analysis was performed employing the Bonferroni Correction and thus setting the significance level at *p* = 0.00125 (40 analyses). Regarding the associations between oocyte characteristics, behavior during ICSI and Z-Score, the significance level was set at *p* = 0.0025 (20 analyses). Regarding associations between oocyte characteristics, behavior during ICSI and embryo quality, the significance level was set at *p* = 0.001 (50 analyses). Finally, regarding associations between oocyte characteristics, oocyte behavior, and the probability to result to post-ICSI oocytes and embryos selected to be discarded, the significance level was set at *p* = 0.005 (10 analyses).

## Results

**Figure 1:**
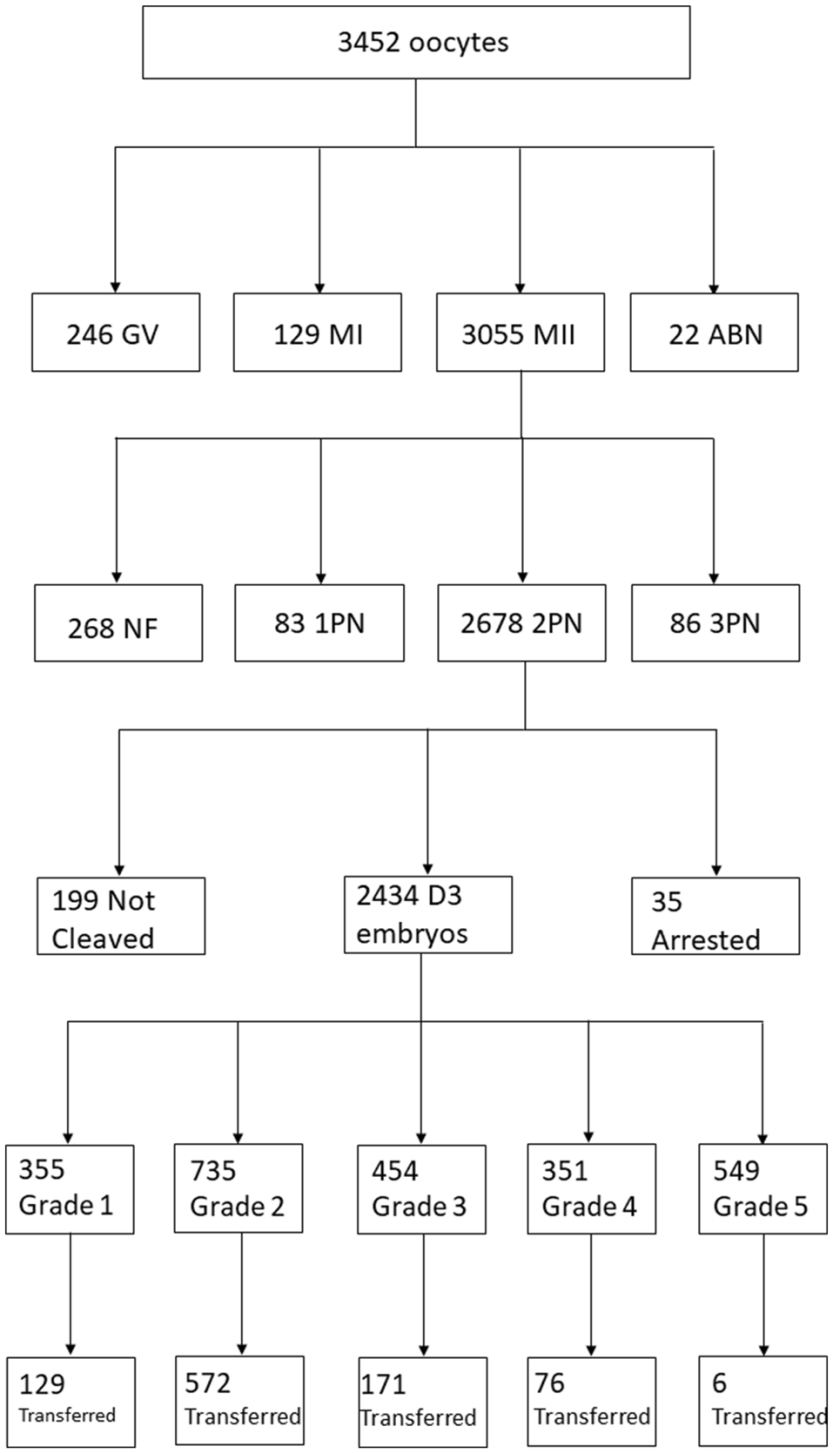
Workflow chart regarding patient and oocyte characteristics.

**Figure 2:**
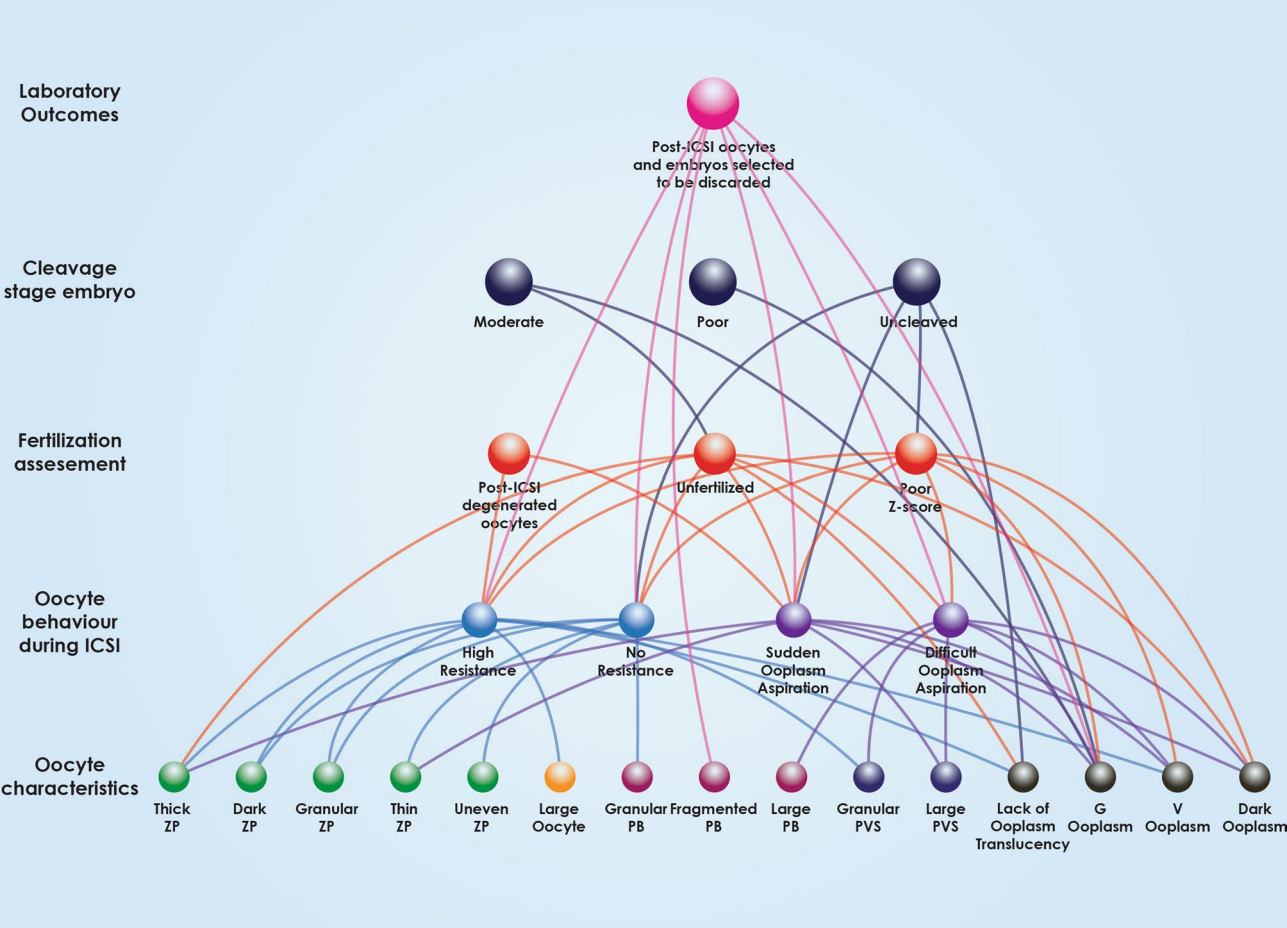
All-inclusive associations between oocyte characteristics, behavior during ICSI, fertilization, Z-Score, embryo grading and post-ICSI oocytes and embryos selected to be discarded.

The mean age of our patients was 36.45 (± 1.65), ranging from 31 to 39. Patient’s age and hormonal levels are presented in Table 1. Number of oocytes retrieved, their maturity status, information about the zygotes and day 3 embryo quality are also presented in Table 1. Embryo quality of the transferred embryos is presented in Table 2. A workflow chart is presented in Fig. 1. Positive hCG rate for our patients 28.30% (135/477), and respectively live birth rate was noted for 21.17% (101/477) of the recruited study sample. Graphical representation of the statistically significant-following Bonferroni Correction-associations observed is provided in Fig. 2. Supplementary Table 2 provides data on the absolute number of oocytes featuring a certain studied morphological characteristic, along with the observation frequency of the said characteristic, for both the overall cohort of oocytes, as well as for the ones corresponding to transferred embryos.

**Table 1:** Patient characteristics and embryological data.

	Total cohort
Female age	36.45 ± 1.45
FSH (mIU/mL)	7.29 ± 1.02
LH (mIU/mL)	4.55 ± 0.83
E_2_ (pg/mL)	2110.99 ± 277.15
AMH (ng/mL)	3.67 ± 0.80
Progesterone (ng/mL)	12.66 ± 3.28
Oocytes retrieved	3452
MII	3055 (88.50%)^a^
MI	129 (3.74%)^a^
GV	246 (7.13%)^a^
Abnormal	22 (0.64%)^a^
2PN	2678 (87.66%)^b^
1PN	83 (2.72%)^b^
3PN	86 (2.28%)^b^
Unfertilized	181 (5.92%)^b^
Degenerated oocytes post-ICSI	87 (2.85%)
Uncleaved	199 (7.43%)^c^
Arrested	35 (1.31%)^c^
Day 3 Grade 1	355 (13.26%)^c^
Day 3 Grade 2	735 (27.45%)^c^
Day 3 Grade 3	454 (16.95%)^c^
Day 3 Grade 4	351 (13.11%)^c^
Day 3 Grade 5	549 (20.50%)^c^

^a^Divided by the total number of oocytes.

^b^Divided by the total number of MII oocytes.

^c^Divided by the total number of 2PN zygotes.

**Table 2:** Grading of embryos transferred.

Day 3 quality	Number of embryos transferred (n = 954)
Grade 1	129 (13.52%)
Grade 2	572 (59.96%)
Grade 3	177 (18.55%)
Grade 4	76 (7.97%)
Grade 5	0 (0%)

### Associations between oocyte and patient characteristics

No association was observed between the levels of AMH, E2, FSH, LH or progesterone and any of the oocyte characteristics. Age was negatively associated with the observation frequency for normal ooplasm (*p* < 0.001) and normal PVS (*p* = 0.001). The observation frequency regarding granular oocytes and granular PVS was positively associated with maternal age (*p* < 0.001). No other statistically significant association was presented.

### Oocyte characteristics and Z-score

The authors decided that Z1 score being associated with the best prognosis should be employed as the reference point in the multinomial regression analysis. A dark ooplasm (*p* < 0.001) was positively associated with fertilization failure. Oocytes that failed to be fertilized presented with a lack of ooplasm translucency (*p* < 0.001). A thick ZP was similarly positively associated with non-fertilized oocytes (*p* < 0.001). The specific odds ratio along with their confidence intervals when compared to the corresponding normal characteristic are presented in Table 3.

**Table 3:** Odds ratio (and 95% CI) of associations between oocyte characteristics and Z-Score.

	Unfertilized	Z3	Z4
Dark ooplasm	11.213 (3.02–41.62)	9.57 (2.96–30.89)	15.72 (4.28–57.68)
Granular ooplasm	NS	9.27 (4.92–17.48)	4.99 (2.40–10.35)
Vacuolated ooplasm	NS	NS	22.69 (5.61–91.70)
Lack of ooplasm translucency	9.77 (4.33–22.04)	NS	NS
Thick ZP	13.13 (4.94–38.38)	NS	NS

### Oocyte characteristics and embryo grading

The authors decided that grade 1 score being associated with the best prognosis should be employed as the reference point in the multinomial regression analysis. Uncleaved embryos were correlated with lack of ooplasm translucency (*p* = 0.001). Embryos graded as 3, 4 and 5 were associated only with ooplasm granularity (*p* < 0.001).

### Oocyte characteristics and oocyte behavior during ICSI

#### Oocyte characteristics and penetration difficulty

A high resistance during ICSI penetration (HR) was associated with lack of ooplasm translucency (*p* < 0.001), an extensively granular PVS (*p* < 0.001) and abnormalities of zona pellucida, namely thick, dark or granular ZP (*p* < 0.001). The large oocyte size (*p* < 0.001), the vacuolated (*p* < 0.001) ooplasm, the extensively granular PB (*p* < 0.001) or the thin, dark, granular or uneven ZP (*p* < 0.001) were statistically significantly associated with a lack of resistance during ICSI (NR). Associations between oocyte characteristics and penetration difficulty are presented in Table 4.

**Table 4:** Odds ratio (and 95% CI) of associations between oocyte characteristics and oocyte behavior during ICSI.

	High resistance	No resistance	Sudden ooplasm aspiration	Difficult ooplasm aspiration
Large oocyte size	NS	10.40 (3.27–33.10)	NS	NS
Dark ooplasm	NS	NS	14.75 (7.72–29.89)	16.03 (7.45–34.48)
Granular ooplasm	NS	NS	4.21 (2.68–6.59)	8.57 (5.50–13.34)
Vacuolated ooplasm	NS	7.59 (3.12–18.43)	16.34 (7.52–35.48)	12.24 (5.11–29.19)
Lack of ooplasm translucency	3.13 (1.77–5.33)	NS	NS	NS
Granular PVS	10.53 (6.27–26.60)	NS	NS	3.40 (2.07–5.57)
Large PVS	NS	NS	29.85 (7.57–117.637)	16.07 (3.39–76.05)
Thick ZP	13.39 (6.81–29.02)	NS	3.62 (1.85–7.07)	NS
Thin ZP	NS	111.13 (10.84–1139.12)	12.48 (3.53–44.12)	NS
Uneven ZP	NS	331.25 (30.89–3551.65)	NS	NS
Dark ZP	83.48 (30.68–227.09)	23.51 (7.73–71.48)	NS	NS
Granular ZP	10.27 (4.81–21.91)	12.10 (3.63–40.31)	NS	NS
Large PB	NS	NS	NS	53.18 (5.86–481.97)
Granular PB	NS	33.33(9.36–118.71)	NS	NS

*NS* not significant.

#### Oocyte characteristics and ooplasm aspiration

A difficult ooplasm aspiration was positively associated with a dark, granular or vacuolated ooplasm (*p* < 0.001), a granular or large PVS (*p* < 0.001) or a large PB (*p* < 0.001). A sudden ooplasm aspiration was positively associated with a granular, vacuolated or dark ooplasm (*p* < 0.001), as well as, a granular (*p* < 0.001) or large (*p* < 0.001) PVS and a thick (*p* < 0.001) or thin (*p* < 0.001) ZP. Associations between oocyte characteristics and ooplasm aspiration are presented in Table 4.

#### Oocyte behavior during ICSI and Z-score

Oocytes presenting with No Resistance (NR) were more likely to be graded as Z4 (*p* < 0.001) or fail to be fertilized (*p* < 0.001) when compared to normal resistance. Oocytes presenting with High resistance (HR) were more likely to fail to be fertilized or be categorized as degenerated oocytes post-ICSI, when compared to normal resistance (*p* < 0.001) or to be graded as Z2 or Z4 (*p* < 0.001). In cases where ooplasm aspiration was difficult the oocytes were more likely to fail to be fertilized or to be graded as Z2 or Z3 (*p* < 0.001). In cases where the ooplasm aspiration was sudden the oocytes were more likely to fail to be fertilized (*p* < 0.001) be categorized as degenerated oocytes post-ICSI (*p* < 0.001) or to be graded as Z3 or Z4 (*p* < 0.001).

#### Oocyte behavior during ICSI and embryo grading

The authors decided that grade 1 score being associated with the best prognosis should be employed as the reference point in the multinomial regression analysis. Uncleaved embryos were correlated with NR (*p* < 0.001) as well as sudden ooplasm aspiration (*p* = 0.001).

### Oocyte characteristics, oocyte behavior and post-ICSI oocytes and embryos selected to be discarded

A total of 1200 post-ICSI oocytes and embryos were selected to be discarded during the 477 IVF cycles. The post-ICSI oocytes and embryos selected to be discarded correspond to 83 1PN embryos, 86 3PN embryos, 181 unfertilized and 87 degenerated post-ICSI oocytes, 199 uncleaved, 35 arrested and 549 grade 5 embryos. Regarding the oocyte’s characteristics, oocytes with granular ooplasm were associated with higher probabilities of resulting to post-ICSI oocytes and embryos selected to be discarded (*p* = 0.001). Similarly, oocytes presenting with a fragmented polar body were associated with higher probabilities of resulting to an embryo selected to be discarded (*p* < 0.001). Both sudden and difficult ooplasm aspiration were associated with higher probabilities of resulting to post-ICSI oocytes and embryos selected to be discarded (*p* < 0.001). Similarly, either high or no resistance during ICSI were associated with higher probabilities of developing to post-ICSI oocytes and embryos selected to be discarded (*p* < 0.001).

## Discussion

Identifying oocyte characteristics that may be related to the oocyte’s behavior during ICSI, and subsequent z-score classification at fertilization evaluation is undoubtably of value. This may in turn enable the possibility that the outcome of ICSI procedure, fertilization rate and zygote potential may be predicted by an early oocyte evaluation. It is this incentive that served as the driver for this study. Deciphering on “how” these characteristics lead to specific outcomes should be the focus of further studies employing basic research in unveiling the molecular pathways involved. Being able to predict the oocytes’ attitudes during ICSI and subsequent zygote dynamic and embryo quality, may prove particularly beneficial especially for countries where the law permits insemination of a certain restricted number of oocytes. For these cases, a preliminary selection of oocytes based on characteristics that have predictive value in regards to an improved performance of the oocyte within an ICSI cycle is a prerequisite. Is it possible that information we collect on the oocyte on Day 0 prior to submitting it to insemination represents a key factor in embryo selection^9^? This scenario has been revisited throughout the years^10^. Nonetheless, oocyte morphological assessment may be of value strictly in the single culture scenario. Is the single embryo culture scenario-outside the scope of TLM-relevant in 2020? The majority of clinical embryology research studies opt for group embryo culture^40^, being associated with higher blastocyst formation rate^41,42^, albeit presenting no statistically significant difference regarding clinical pregnancy rates^41^. In the era of TLM, single embryo culture has emerged again^43^. Even though outside the context of TLM, group culture is favored, nonetheless, conventional single embryo culture should be the employed method of choice especially in research protocols, if not in routine clinical practice.

Abnormal oocyte morphology has been associated with different negative outcomes in an ICSI cycle^9,44–46^. Interestingly, no particular characteristic has been associated with specific outcomes. The lack of a universal oocyte grading system along with the numerous morphological characteristics that may be assessed constitute evaluation of the oocyte morphology cost-intensive. Analysis has been performed regarding the respective associations between oocyte characteristics and their behavior during ICSI with resulting post-ICSI oocytes and embryos. This is performed in an effort to provide a clearer take-home message not only regarding specific embryo quality, but from the perspective of a less vague and more defined IVF laboratory outcome-the negative one. The rational in investigating respective associations strictly with negative laboratory outcomes is outlined herein. The endpoint associations attempted could in theory pertain to three categories of post-ICSI oocytes and resulting embryos in the IVF laboratory .Category 1 refers to embryos selected for embryo transfer. Category 2 refers to embryos selected for cryopreservation. Category 3 refers to post-ICSI oocytes and embryos selected to be discarded. Category 1 includes embryo transfer embryos that are comparatively the best quality embryos of the cycle, but not necessarily of good quality if the cycle entails average or poor quality of embryos as a whole. Category 2 includes good quality embryos selected for cryopreservation that are however comparatively not the best quality embryos of the cycle, since those have been selected for embryo transfer. Finally, category 3 includes embryos selected to be discarded on the grounds of poor quality, along with post-ICSI oocytes classified as unfertilized, abnormally fertilized and degenerated. Evaluating the suitability of the three categories in decision-making regarding drawing conclusive associations with oocytes’ characteristics and ICSI behaviour and indicating conclusive relationships, it appears that it is only category 3 that can provide certainty in established clear associations, simply attributed to the fact that this category includes uniformly poor quality elements related to a negative IVF laboratory outcome automatically extending to a negative clinical outcome. On the contrary, category 1 and 2 include a less uniform, and a more heterogeneous group of embryos, as ET or cryopreservation does not necessarily include the best quality embryos, or the best quality embryos of the cycle respectively. To provide a more robust and unbiased perspective the analysis conducted regarding associations with post-ICSI oocytes and embryos selected to be discarded includes strictly unfertilized, degenerated and abnormally fertilized oocytes post-ICSI, along with uncleaved zygotes, arrested embryos and grade 5 embryos of extensively poor quality. Hence, associations presented herein report on identifying oocyte characteristics and ICSI behavior patterns related with any negative IVF laboratory outcome and subsequently lack of achieving implantation, clinical pregnancy live-birth following an ET.

The aim of this study is to provide practitioners with an indication as to which oocyte characteristics convey the highest weight factor, and hence deserve to be prioritized in evaluation. It should be noted that this study has indicated strictly negative associations. This study cannot attempt to provide data on positive predictors. This is attributed to the design of the statistical analysis, which included normal oocyte characteristics and normal ICSI behavior, along with the top zygote and embryo quality-being regarded as the best prognosis features-as reference points. Due to this no positive predictors could be evaluated. Further to that, a negative outcome may be the result of a single abnormality, whereas a positive outcome requires the synergy of numerous factors.

### Interpretation of results

According to our results, abnormal oocyte characteristics exert a substantial negative role regarding both oocyte behavior during ICSI, Z-Score and embryo quality. All abnormal ooplasm characteristics were equally associated with either types of abnormal ooplasm aspiration namely sudden or difficult. Nonetheless, results were inconclusive with respect to the abruptness or difficulty of the aspiration as no particular abnormal feature of the oocyte appeared to be related to one or the other. The presence of a dark cytoplasm was associated with unfertilized oocytes, Z3 and Z4 scores. This is in accordance to literature as dark cytoplasm has been associated with lower fertilization rates^15^ and embryos of poorer quality^47^. Granular ooplasm was associated with poorer prognosis Z-Scores, namely Z3 and Z4 moderate and poor cleavage stage embryo quality and were more likely to be discarded at any developmental stage. Granularity of the ooplasm is correlated in literature with lower implantation potential and embryos of inferior quality^48,49^. Fertilization failure was not associated with ooplasm granularity in this study, an observation supported by part of published data^49^ and contradicted by other studies^15^. The presence of vacuoles was associated with Z4 scores, supporting the observation that vacuoles have been correlated with a lower blastocyst formation rate^3^. Lack of ooplasm translucency was associated with unfertilized post-ICSI oocytes, high resistance during ICSI, as well as cleavage failure. The association of lack of ooplasm translucency with unfertilized post-ICSI oocytes has similarly been observed in animal studies, and more specifically in bovine^50^. Although lack of ooplasm translucency has not been associated in literature with specific adverse effects, it has been voiced that a good quality mature oocyte is defined as an oocyte with clear, moderately granular ooplasm^51^. It may be possible that lack of oocyte translucency could signify a point of different maturity status on the cellular level of an oocyte. It should be highlighted that according to the results sourced herein, granularity of the ooplasm appears to be associated with poor prognosis in the IVF laboratory and correspond to impaired post-ICSI oocytes and embryos that may be destined to be discarded. This observation may prove to be of significance when employed in a future prediction model.

The morphological characteristics of the oocyte were also associated with the behavior of the oocyte during ICSI. A thick ZP was associated-as expected-with a HR in ICSI penetration whereas the thin ZP was associated with NR, supporting current literature observations^52^. A thick ZP was further associated with unfertilized post-ICSI oocytes, an observation that is in agreement with published data^53^. Sudden ooplasm aspiration was also associated with a thin ZP. It appears that size abnormalities of the zona were predictive of sudden ooplasm aspiration, and are not related to difficult ooplasm aspiration, whereas texture-namely granularity and darkness-abnormalities of the ZP were predictive of either abnormal types of penetration resistance during ICSI. No direct association between ZP size or texture with Z-Score or day 3 embryo quality was observed.

Large PVS has been associated with sudden oolemma breakage in literature^10^. A granular PVS may be associated with HR and difficult ooplasm aspiration. Extrapolating on these results, could PVS be of interceptive nature and thus affect difficulty of penetration during ICSI? Both abnormalities of the PVS have been associated with poorer implantation and pregnancy rates^54^.

The polar body’s granularity was associated with NR. A large PB size was associated with a difficult ooplasm aspiration during ICSI. The large PB has been associated in literature with a lower fertilization rate^55^, which has not been observed in the present study. Oocytes with fragmented PB were associated with higher probabilities of being discarded at any developmental stage. Polar body fragmentation has been associated in literature with lower normal zygote formation rate and with embryos of poorer quality^56^. Similar observations have been reported in studies including mice^57^.

Behavior of the oocytes during ICSI was associated with fertilization outcome, Z-Score and cleavage rate. Both HR and NR were associated with either unfertilized post-ICSI oocytes or Z4. NR during ICSI was associated with cleavage failure. HR during ICSI was also associated with Z2. HR was associated with degenerated post-ICSI oocytes. This is in accordance to literature. As observed by Danfour and colleagues oocytes featuring high resistance during ICSI presented with statistically significantly lower normal fertilization rates when compared to normal penetration resistance. High penetration resistance, leads to a persistent funnel during the injection, which has been associated with intrinsically ooplasmic effects.

Abnormal ooplasm aspiration sudden or difficult was associated with unfertilized post-ICSI oocytes and degenerated oocytes post-ICSI. Apart from that, a difficult ooplasm aspiration was associated with either Z2 or Z3, whereas the sudden ooplasm aspiration was associated with Z3 or Z4. Sudden ooplasm aspiration was associated with cleavage failure. These results present a pattern in which the abnormal penetration status is directly associated with fertilization failure. These results are concordant with current bibliography^23,58^. Sudden oolemma breakage has been suggested to be attributed to fragile oocytes^22,23^. Oocyte fragility and elasticity are topics that merit further investigation. Softening of the ZP during oocyte maturation and its hardening following fertilization is the physiological pathway^59^. More studies are needed in order to investigate and delineate the reasons behind the divergence from this pathway towards a harder ZP that may lead to a sudden or difficult oolemma breakage. As observed in literature, embryos deriving from oocytes with difficult oolemma breakage, may require assisted hatching in order to improve clinical pregnancy and live-birth potential (Ebner et al.^11^).

**Figure 3:**
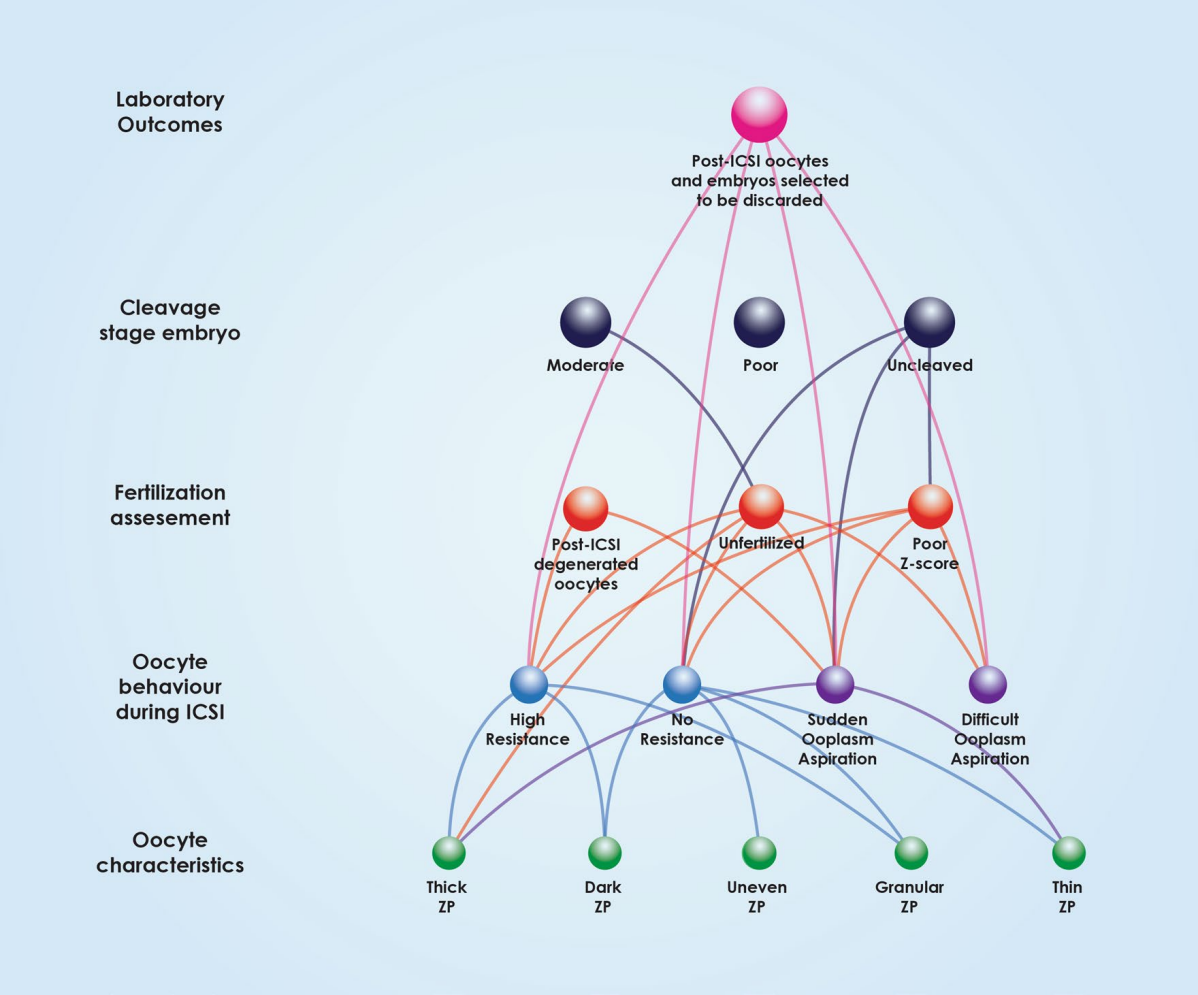
Associations between polar body, perivitelline space characteristics, oocyte size, behaviour during ICSI, fertilization, Z-Score, embryo grading and post-ICSI oocytes and embryos selected to be discarded.

**Figure 4:**
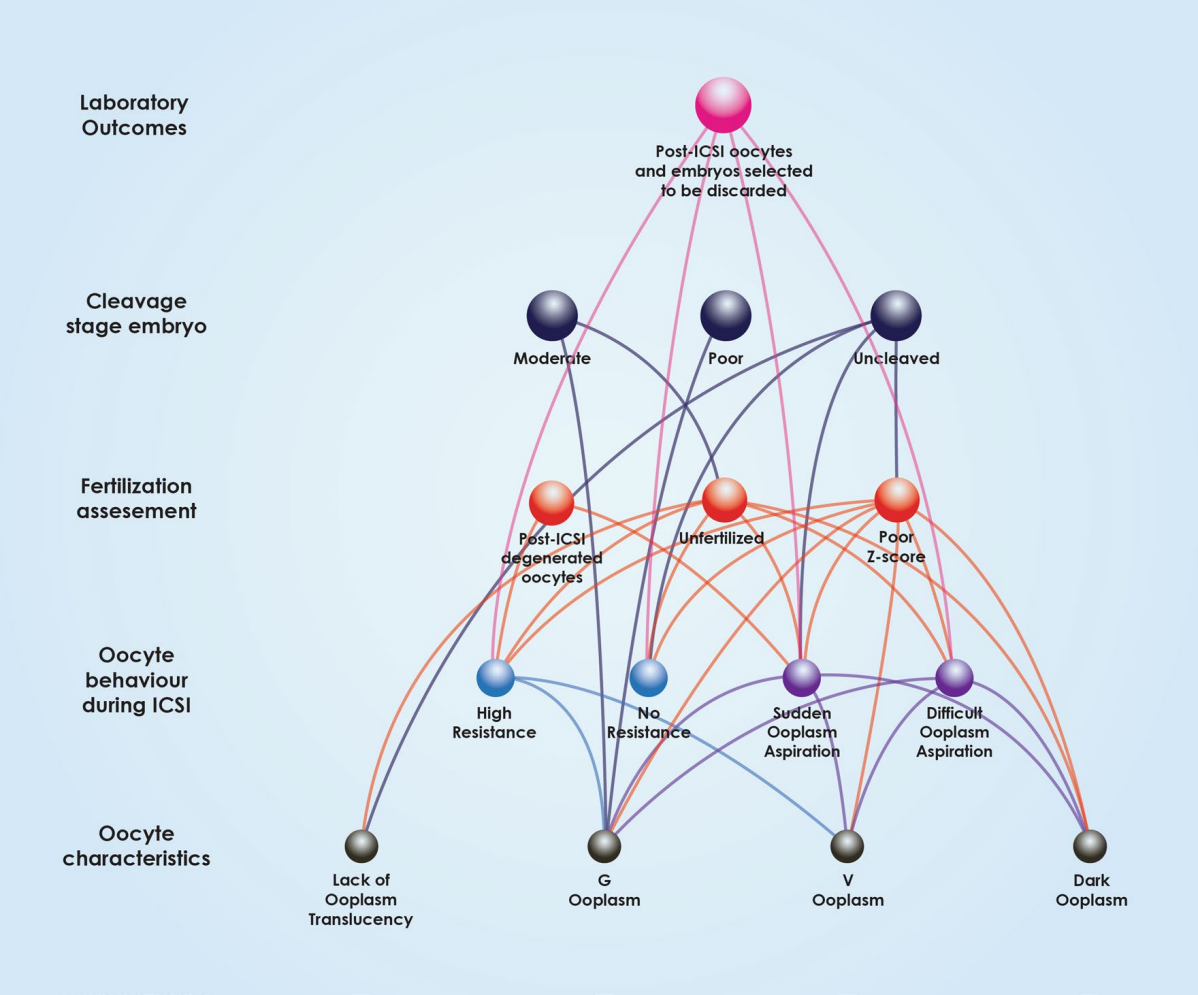
Associations between Zona Pellucida characteristics, behaviour during ICSI, fertilization, Z-Score, embryo grading and post-ICSI oocytes and embryos selected to be discarded.

**Figure 5:**
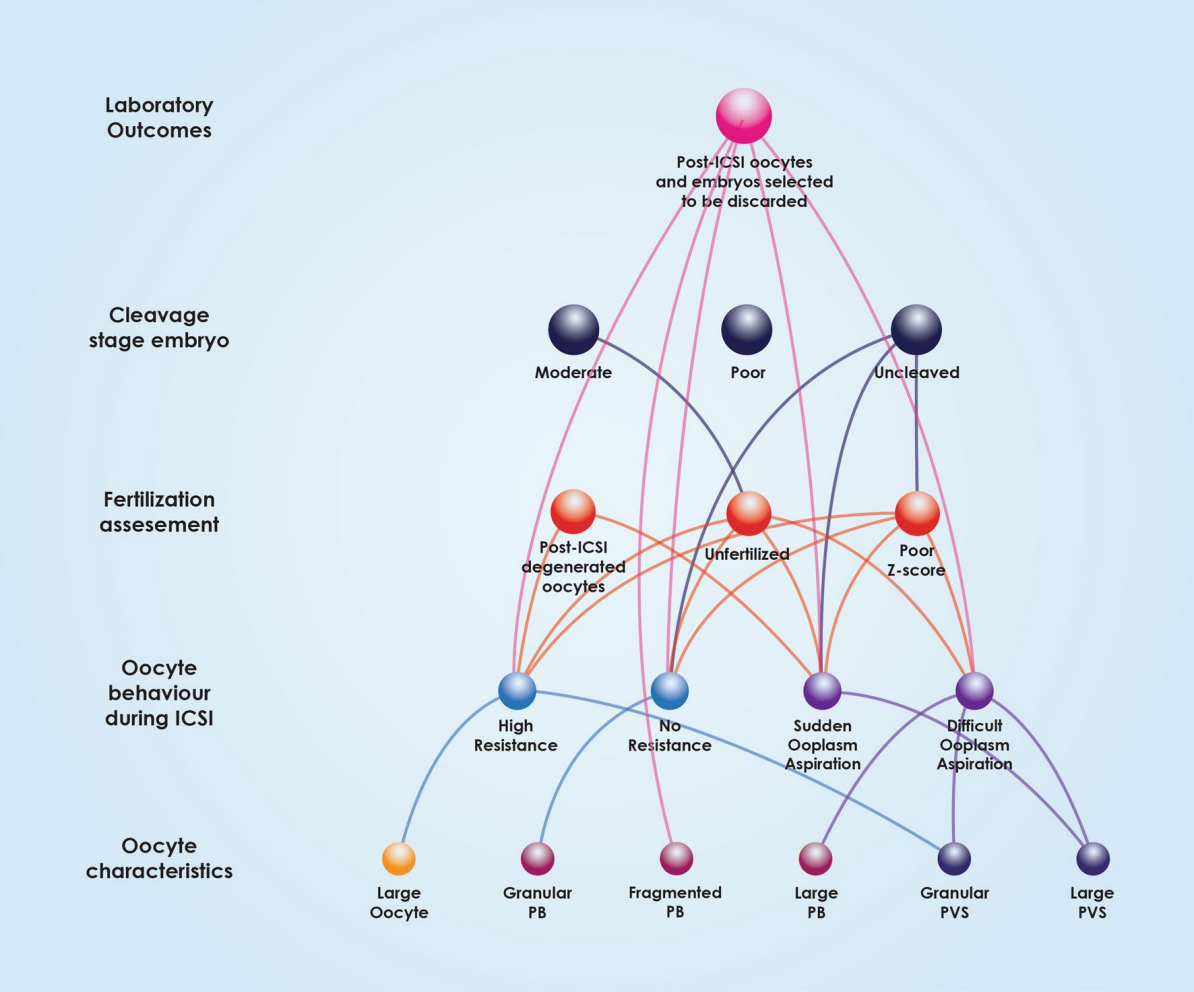
Associations between ooplasm characteristics behaviour during ICSI, fertilization, Z-Score, embryo grading and post-ICSI oocytes and embryos selected to be discarded.

Multiple associations between oocyte characteristics, oocyte behavior during ICSI, fertilization assessment and embryo quality are comprehensively outlined in Fig. 2. This Figure indicates the various “levels” of associations, from the level of oocyte morphological characteristics, to ICSI behavior, to the level of fertilization, leading to cleavage and finally the level of clinical outcome. This Figure presents the directness of associations regarding the various levels also corresponding to various time-points of assessment and developmental milestones, from the oocyte to day 3 embryo quality. As presented in Fig. 3, oocyte characteristics regarding the PVS, PB and oocyte size have only been associated with the next “level” of observations corresponding to the time-point of the oocyte’s behavior during ICSI. ZP characteristics depicted in Fig. 4 appear to be mainly associated with the level of observations corresponding to ICSI behavior, with the exception of thick ZP, which appears to be directly associated with fertilization failure bypassing and irrespectively of the oocyte’s behavior during ICSI. Ooplasm characteristics represented in Fig. 5, were associated with all levels and time-points of observation, bypassing and irrespectively of the in-between developmental stages of observation.

### Limitations

A number of associations that were initially regarded as statistically significant were disregarded following the sensitivity analysis (Supplementary Table 3). The authors opted to employ Bonferroni correction due to the high number of variables employed in the statistical analysis, in order to reduce multiple associations bias. These correlations may have not been disregarded if the sample size was larger. This was performed to reduce possible confounding. As it can be observed from Supplementary Table 2 the “normal” characteristic was the most common observation. This considerable difference between the frequencies of observations on studied parameters favouring the observation “normal characteristic” was anticipated in light of the study’s setting being a clinical practice IVF laboratory. With regards to the most frequently observed abnormal oocyte morphology characteristics, the study indicated fragmented polar body with a frequency of observation 28.25%, followed by lack of ooplasm translucency at 26.28% and ooplasm granularity at 13.97%. The overall smaller sample size observed for each abnormality as well as the fact that a number of them were associated with fertilization and/or cleavage failure, may hinder the possibilities of observing a statistically significant correlation with IVF outcome.

Double ET, which was performed due to the clinical setting of this study, stands as a major limitation in the study design as it renders it impossible to know which of the two embryos implanted, and draw respective associations. As a result, this study does not allow for any conclusion to be extracted by relying on associations of any of the characteristics studied with implantation failure or success. Clinical observational studies may often be compromised in the name of standard clinical practice.

As this was an observational study, the authors employed day 3 grading as the criterion for embryo transfer, and oocyte characteristics did not play a part in decision making. This was opted in accordance to the standing protocol of the clinic and may present as a limitation when examining the effects of oocyte characteristics and their behaviour during ICSI. A major limitation in the study is the lack of time-lapse microscopy which would ensure a dynamic evaluation at the zygote stage. The static evaluation of the Z-score employed herein presents with limitations considering that the behaviour of PNBs within PNs represents a dynamic process^27^. The moderate sample size, as well as the fact that the study was carried out in a single center should be regarded as further limitations of the study.

What should be noted is that in an effort to provide possible explanations on our findings and report on published studies that have focused on oocyte characteristics and implications, it became apparent that the majority of articles document experience originating from previous decades-in fact sometimes as far as 20 years back. It may be of interest that subjects such as oolemma breakage during ICSI were thoroughly studied back in the ‘90 s however, no further entries have been noted in the literature. The injection technique is characterized by high variation (Palermo et al.^23^), which may justify the variant description of the oocyte’s ICSI behavior. This is in turn reflected in numerous publications describing diverse oolemma breakage evaluations, coupled by the subsequently differently defined ooleemma breakage types^22–25^. Respective terminology is accordingly different. From stabbing, pricking, penetrating, manoeuvring, and invagination types, funnel shapes, shifts, depth and curves, to suction, and volume of cytoplasm aspirated, the oolemma response to injection is subject to various descriptions^24,25,60^. Nonetheless, the common denominator is that oolemma breakage, and subsequent ooplasm aspiration types depend on characteristics of the membrane’s structure, rigidity, elasticity and fragility along with the cytoplasm’ maturity status^38,58,61^.

### Future research recommendations

In the era of TLM identifying parameters related to the oocyte implicated earlier in development and preceding the creation of the embryo is of critical importance. Acknowledging and indicating their predictive value in improving the efficiency of decision-making on the optimal embryo(s) to be transferred may be priceless. Published studies so far have indicated a correlation between the size of the oocyte and time to separation of the 2nd polar body (tBP2), time to 5 cells (t5) and time to 8 cells (t8)^46^. The authors strictly compared diameters of the whole oocyte, the ooplasm, the ZP, the PVS and of the PB. An earlier study by the same group observed no statistically significant difference between the morphologically normal and abnormal oocytes, though it must be noted that there was no subgroup analysis regarding each of the abnormal parameters^46^. Undoubtedly, the advent of TLM has enabled collection of detailed images dynamically associated with a clinical endpoint. Albeit morphokinetics drive the way forward, further data may be required in supporting their beneficial role^62^ leading to the conclusion that static observations may still have a lot to offer. It may be possible that an integration of both observation types, with clearer cut-off points and parameters, will improve clinical pregnancy and live birth rates. It may be possible that inclusion of pre-fertilization observations will enhance performance of a future prediction model. The present study aims to contribute towards the development of future prediction models, by highlighting the importance of a time-point that is not assessed by TLM or employed in the majority of Artificial Intelligence (AI) algorithms. Perhaps the recording and the evaluation by AI should be initiated at a point prior to fertilization. It may be of significance to include both static and dynamic oocyte data from pre-fertilization in a future AI prediction model. The strength of AI relies on the employment of a great number of parameters and associations performed employing multiple hidden layers. The implementation of the parameters observed as significant in the present study, may require development of novel devices-perhaps even enabling evaluation of oocyte behavior during ICSI-as well as great computing power. However, an all-inclusive AI model may represent-in the not too distant future-the critical point in enhancing IVF success rates.

Can day 0 of an ICSI cycle be indicative of the outcome? Despite thorough documentation of data regarding oocyte parameters, only extrapolations and assumptions may be attempted herein. Is connecting the dots on the itinerary from oocyte, to embryo, through the ICSI route enough? The authors purposefully refrain from proceeding to forming hypotheses and drawing conclusions regarding the results of this strictly observational study that reports on findings. Identifying the connections is only part of the equation. It is understanding the exact route that will convey safety in deciding the weighing factor of all the parameters studied. More basic research is required in clinical embryology employing human embryos. Investigation of the molecular pathways involved and related to specific characteristics, certain oocyte behavior and developmental dynamic is imperative. Such studies may provide the understanding required in order to enhance IVF cycles success rates, and even extent to an improved obstetric, perinatal outcome and pediatric follow-up. The scenario of whether the answer lies with the oocyte and its behavior during ICSI may still prove of interest. Although associations between oocyte characteristics and the different laboratory outcomes studied have been identified, it is not always an “all or nothing” phenomenon. Therefore the question that needs to be answered is “Will a lower quality embryo on day 3 or day 5 be selected over an excellent embryo based on specific oocyte characteristics?” To answer this, it may be of interest to perform an interventional study, in which in one group the decision for embryo transfer should be based on oocyte characteristics and respective behavior during ICSI, while in the other group the decision for ET should be based solely on the embryo morphology on the day of ET. It is possible that this study design may not even provide the final answer, which may lie in developing a grading system or prediction model that will include data representing both oocyte and embryo characteristics of these characteristics.

## Conclusion

According to the results of our study, it appears that various abnormal oocyte characteristics and abnormal behavior during ICSI negatively influence the laboratory outcomes of an ICSI cycle. It may be possible that several characteristics that have not presented with a direct association may exert an indirect influence on the laboratory outcomes. This indirect influence may hint that the developmental potential of the pre-implantation embryo should not be represented by a linear route with specific parameters influencing the endpoint, but should be rather described as a multi-layered network with the developmental checkpoints remaining undefined. It is identifying these checkpoints that signify different developmental events and connecting them to either morphological or morphokinetical parameters that may be the missing piece of the puzzle. The message this study aims to convey is that oocyte granularity, as well as polar body fragmentation, along with type of ooplasm aspiration appear to exert a negative direct effect, as evidenced by the increase in the number of post-ICSI oocytes and embryos selected to be discarded. If these observations are validated by other studies, it may be possible that future prediction models may rely to assessing more time-points in order to improve ICSI cycle outcome prediction.

## Supplementary information

Supplementary Tables.
