# Supplementary material for: Evaluating the value of day 0 of an ICSI cycle on indicating laboratory outcome
Source: Sci Rep. 2020 Nov 9;10:19325. doi: 10.1038/s41598-020-75164-9 (PMC7653966; doi:10.1038/s41598-020-75164-9)
Supplement: Supplementary file 1 — Supplementary Tables. [file 41598_2020_75164_MOESM1_ESM.docx]

**Evaluating the value of day 0 of an ICSI cycle on indicating laboratory outcome**

**Maziotis E^1,2^, Sfakianoudis K.^3^, Giannelou P^1,3^, Grigoriadis S_­_^1^, Rapani A^1^, Tsioulou P^1^, Nikolettos K^4^, Pantou A^3^, Tiptiri-Kourpeti A^4^, Koutsilieris M^1^, Asimakopoulos B^2^, Nikolettos N^2^, Pantos K^3^, Simopoulou M^1^.**

*^1^Department of Physiology, Medical School, National and Kapodistrian University of Athens, Athens, Greece*

*^2^Department of Physiology, Medical School, Democritus University of Thrace, Alexandroupoli, Greece*

*^3^ Centre for Human Reproduction, Genesis Athens Clinic, Athens, Greece*

*^4^ Assisted Reproduction Unit of Thrace “Embryokosmogenesis”, Alexandroupoli, Greece*

Short Title: Predictive value of Day 0 in an ICSI cycle

Keywords: ICSI, oocyte morphology, Z-Score, oocyte behavior, live birth

Supplementary Table 1: Z-Score as developed by Scott and colleagues (Scott et al., 2000)

| Scoring | Description |
| --- | --- |
| Z1 | Equal number of nucleoli (3-7) aligned at the pronuclear junction |
| Z2 | Equal numbers and sizes of nucleoli (3-7), equally scattered in the two nuclei |
| Z3 | Equal numbers of nucleoli of equal sizes in the same nuclei but with one nucleus having alignment at the pronuclear junction and the other with scattered nucleoli  OR  Unequal number and/or size of nucleoli |
| Z4 | Pronuclei not aligned  OR  Pronuclei of different sizes  OR  Pronuclei not located in the central part of the zygote |

Supplementary Table 2: Observational frequency of oocyte characteristics and ICSI behavior

|  | Total Cohort (n=3055) | Embryos Transferred (n=954) |
| --- | --- | --- |
| **CoCs** |  |  |
| Normal | 2750 (90,02%) | 892 (93,50%) |
| Abnormal | 305 (9,98%) | 65 (6,81%) |
| **Oocyte Shape** |  |  |
| Normal | 2813 (92,07%) | 902 (94,55%) |
| Distorted | 55 (1,80%) | 11 (1,15%) |
| Ovoid | 97 (3,18%) | 11 (1,15%) |
| Uneven | 90 (2,94%) | 20 (2,10%) |
| **Oocyte Size** |  |  |
| Normal | 2799 (91,62%) | 930 (97,48%) |
| Small | 169 (5,53%) | 10 (1,05%) |
| Large | 87 (2,85%) | 14 (1,47%) |
| **Ooplasm Translucency** |  |  |
| Yes | 2252 (73,72%) | 765 (80,19%) |
| No | 803 (26,28%) | 189 (19,81%) |
| **PVS** |  |  |
| Normal | 2279 (74,60%) | 781 (81,87%) |
| Large | 37 (1,21%) | 10 (1,05%) |
| Small | 110 (3,60%) | 28 (2,94%) |
| Granular | 544 (17,81%) | 125 (13,10%) |
| Absent | 85 (2,78%) | 10 (1,05%) |
| **Ooplasm** |  |  |
| Normal | 1920 (62,85%) | 635 (66,56%) |
| Dark | 371 (12,14%) | 77 (8,07%) |
| Granular | 427 (13,97%) | 177 (18,55%) |
| Vacuolated | 337 (11,03%) | 65 (6,81%) |
| **Polar Body** |  |  |
| Normal | 2010 (65,79%) | 818 (85,74%) |
| Fragmented | 863 (28,25%) | 99 (10,38%) |
| Flat | 9 (0,29%) | 7 (0,73%) |
| Granular | 71 (2,32%) | 12 (1,26%) |
| Large | 34 (1,11%) | 9 (0,94%) |
| Small | 68 (2,23%) | 9 (0,94%) |
| **ZP size** |  |  |
| Normal | 2668 (87,33%) | 739 (77,46%) |
| Thick | 238 (7,79%) | 123 (12,89%) |
| Thin | 149 (4,88%) | 82 (8,60%) |
| **ZP Texture** |  |  |
| Normal | 2401 (78,59%) | 805 (84,38%) |
| Uneven | 156 (5,11%) | 29 (3,04%) |
| Granular | 241 (7,89%) | 54 (5,66%) |
| Dark | 250 (8,18%) | 63 (6,60%) |
| Vacuolated | 7 (0,23%) | 3 0,31%) |
| **Penetration Difficulty** |  |  |
| Normal | 2098 (68,67%) | 684 (71,70%) |
| HR | 589 (19,28%) | 190 (19,92%) |
| NR | 368 (12,05%) | 80 (8,39%) |
| **Oolemma Breakage** |  |  |
| Normal | 1744 | 631 |
| Difficult | 559 | 167 |
| Sudden | 752 | 156 |

Supplementary Table 3: Associations with p-value < 0.05 discarded due to Bonferroni correction

| Outcome | Feature | p-value |
| --- | --- | --- |
| Fertilization Failure | Distorted shape | 0.020 |
|  | Granular ooplasm | 0.018 |
|  | Thin ZP | 0.014 |
|  | Dark ZP | 0.020 |
|  | Uneven ZP | 0.047 |
| Z3 | Vacuolated Ooplasm | 0.015 |
|  | NR | 0.038 |
| HR | Abnormal COC | 0.034 |
|  | Small oocyte size | 0.042 |
|  | Granular ooplasm | 0.026 |
|  | Dark ooplasm | 0.007 |
|  | Vacuolated ooplasm | 0.020 |
|  | Granular PB | 0.048 |
|  | Thin ZP | 0.009 |
| NR | Distorted shape | 0.010 |
|  | Uneven shape | 0.032 |
|  | Dark ooplasm | 0.045 |
|  | Granular PVS | 0.011 |
| Sudden Oolemma Breakage | Uneven oocyte size | 0.029 |
|  | Small oocyte size | 0.021 |
|  | Absent PVS | 0.014 |
|  | Granular PB | 0.027 |
| Difficult Oolemma Breakage | Absent PVS | 0.032 |
